# Synthesis and Antiproliferative Activity of Steroidal Thiosemicarbazone Platinum (Pt(II)) Complexes

**DOI:** 10.1155/2015/742592

**Published:** 2015-10-08

**Authors:** Yanmin Huang, Erbin Kong, Chunfang Gan, Zhiping Liu, Qifu Lin, Jianguo Cui

**Affiliations:** ^1^College of Chemistry and Materials Science, Guangxi Teachers Education University, Nanning 530001, China; ^2^Guangxi Colleges and University Key Laboratory of Beibu Gulf Oil and Natural Gas Resource Effective Utilization, Qizhou University, Qizhou 535000, China

## Abstract

Steroidal compounds exhibit particular physiological activities. In this paper, some steroidal thiosemicarbazones platinum (Pt(II)) complexes were synthesized by the condensation of steroidal ketones with thiosemicarbazide using estrone, chenodeoxycholic acid, and 7-deoxycholic acid as starting materials and complexation of steroidal thiosesemicarbazones with Pt(II). The complexes were characterized by IR, NMR, and MS, and their antiproliferative activities were evaluated. The results showed that some steroidal thiosemicarbazones platinum (Pt(II)) complexes displayed moderate cytotoxicity to HeLa and Bel-7404 cells. Thereinto, complex **6** showed an excellent inhibited selectivity to HeLa cells with an IC_50_ value of 9.2 *μ*M and SI value of 21.7. At the same time, all compounds were almost inactive to HEK293T (normal kidney epithelial cells). The information obtained from the studies may be useful for the design of novel chemotherapeutic drugs.

## 1. Introduction

In the late 1960s, anticancer activity of cisplatin was found by Rosenberg [1–4]. Subsequently, cisplatin had become a metal anticancer drug for the treatment of human cancers. But in the process of treatment, cisplatin showed a high toxicity to patients and led to some strong side effects [5]. In accordance with the traditional structure-activity relationship of platinum complexes, the synthesis of new platinum anticancer drugs with the same mode of action has difficulty in achieving a major breakthrough. Because steroid hormones play an important role in the biochemistry of many cancers, a number of steroidal complexes connected to a metal pharmacophore had been designed and synthesized by many research groups, and their physiological activities were evaluated [6–15]. Steroidal metal complexes are transferred to cancer cells by steroidal carrier, selectively accumulated in cancer cells, and combined with specific DNA to reduce the destruction of normal cells. Not only can the metal pharmacophores of steroidal metal complexes enhance DNA damage by its space hinder, but also they play the role of steroid hormones and interfere with cancer cell growth regulating process. Therefore, maintaining and improving the two parts of the activity of the steroidal part and platinum pharmacophore are the key to the molecular design strategy of anticancer steroidal platinum complexes [16–18]. Thiosemicarbazones have received considerable attention since the discovery of their cytotoxic activity against cancer cells and bacteriostatic effects, and the biological properties of thiosemicarbazone complexes are often related and modulated by the metal ion of coordination [19]. Khan and Yusuf [20] and Murugkar et al. [21] investigated the bioactivity of some new steroidal thiosemicarbazones and their Pd(II) or Pt(II) metal complexes and discovered that some compounds had better antibacterial or antineoplastic activity.

In the present study, some novel steroidal thiosemicarbazone platinum (Pt(II)) complexes were synthesized by condensing steroidal ketones with thiosemicarbazide using estrone, chenodeoxycholic acid, and 7-deoxycholic acid as starting materials and complexing steroidal thiosemicarbazone with Pt(II). Their antiproliferative activities against HeLa (human cervical carcinoma), Bel-7404 (human liver carcinoma), and HEK293T (normal kidney epithelial cells) cells were evaluated.

## 2. Materials and Methods

### 2.1. Materials

The sterols were purchased from Sinopharm Chemical Reagent Co., Ltd., Shanghai, China. All chemicals and solvents were of analytical grade from commercial sources. All solvents were used without further purification unless otherwise specified.

### 2.2. Instrumentation and Methods

Melting points were determined on an X_4_ apparatus (Beijing Tech Instrument Co. Ltd., Beijing, China) and were uncorrected. The ^1^H and ^13^C NMR spectra were recorded in CDCl_3_ on a Bruker AV-600 spectrometer at working frequencies 600 and 150 MHz and a Bruker AV-300 spectrometer at working frequencies 300 and 75 MHz, respectively. Chemical shifts are expressed in parts per million (*δ*) values and coupling constants (*J*) in Hertz. Infrared spectra were measured with a Thermo Scientific Nicolet IS-10 Spectrophotometer (Thermo Scientific, America). HREIMS was measured on an Agilent 6210 TOFMS instrument (Agilent Technologies, America). The cell proliferation assay was undertaken by a MTT method using 96-well plates on a MLLTISKAN MK3 analysis spectrometer (Thermo Scientific, Shanghai, China).

### 2.3. Synthesis

Compound** 7** was prepared according to the method of [22] and compound** 12** was prepared following the method of [23].

#### 2.3.1. General Procedure for Preparation of Steroidal Thiosemicarbazone

A mixture of steroidal ketone (1 mmol), thiosemicarbazide (1 mmol), and a few drops of glacial acetic acid in 95% ethanol (30 mL) was stirred at 60–70°C for 10 h. After completion of the reaction, the majority of solvent was evaporated and some water was added to this solution. The mixture was extracted with CH_2_Cl_2_ and the extract was washed with saturated brine, dried with anhydrous sodium sulfate, and evaporated under reduced pressure. The resulting residue was chromatographed on a column of silica gel with a mixture of DCM : methanol (20 : 1) to give steroidal thiosemicarbazone.

*3β-Hydroxyestron-17-thiosemicarbazone (* ***2*** *, L* ^*2*^ *).* White solid, Yield: 78.4%;* m.p.* 254–256°C; IR (KBr) *ν*/cm^−1^: 3419, 3359, 3159, 2927, 1718, 1574, 1464, 1434; ^1^H NMR (600 MHz, DMSO) *δ*: 0.827 (3H, s, 18-CH_3_), 2.567 (1H, dd, *J* = 19.2, 9.0, C6-H), 2.77–2.67 (2H, m, C16-H), 6.436 (1H, d, *J* = 2.4, C4-H), 6.505 (1H, dd, *J* = 8.4, 2.4, C2-H), 7.044 (1H, d, *J* = 8.4, C1-H), 7.397 (1H, s, -NH_2_), 8.046 (1H, s, -NH_2_), 9.030 (1H, s, -NH-), 9.880 (1H, s, -OH); ^13^C NMR (150 MHz, DMSO) *δ*: 17.0 (18-C), 22.9 (11-C), 26.0 (15-C), 26.9 (16-C), 27.0 (7-C), 29.2 (6-C), 33.9 (12-C), 38.0 (9-C), 43.7 (8-C), 44.8 (13-C), 51.9 (14-C), 112.9 (2-C), 115.1 (4-C), 126.2 (1-C), 130.2 (10-C), 137.2 (5-C), 155.1 (3-C), 167.4 (17-C), 178.4 (C=S); HREIMS: *m*/*z* 343.1718 [M+H]^+^ (calcd for C_19_H_25_N_3_OS, 343.1718).

*3β-Acetyloxyestron-17-thiosemicarbazone (* ***5*** *, L* ^*5*^ *).* White solid, Yield: 77.8%;* m.p.* 248-249°C; IR (KBr) *ν*/cm^−1^: 3237, 2930, 1766, 1584, 1516, 1484, 1369, 1147, 936, 813; ^1^H NMR (600 MHz, CDCl_3_) *δ*: 0.907 (3H, s, 18-CH_3_), 2.277 (3H, s, -OCH_3_), 2.40–2.37 (1H, m, C6-H), 2.481 (1H, dd, *J* = 18.0, 8.4, C6-H), 2.90–2.87 (2H, m, C16-H), 6.435 (1H, s, -NH_2_), 6.798 (1H, d, *J* = 2.4, C4-H), 6.845 (1H, dd, *J* = 8.4, 2.4, C2-H), 7.205 (1H, s, -NH_2_), 7.276 (1H, d, *J* = 8.4, C1-H), 8.437 (1H, s, -NH-); ^13^C NMR (75 MHz, DMSO) *δ*: 17.1 (18-C), 21.2 (CH_3_CO), 23.3 (11-C), 26.0 (15-C), 26.4 (16-C), 27.0 (7-C), 29.4 (6-C), 34.0 (12-C), 37.8 (8-C), 44.2 (9-C), 45.1 (13-C), 52.5 (14-C), 118.8 (2-C), 121.7 (4-C), 126.4 (1-C), 137.5 (5-C), 138.1 (10-C), 148.6 (3-C), 167.3 (17-C), 170.0 (COCH_3_), 178.9 (C=S); HREIMS: [M+H]^+^ 408.1736 (calcd for C_21_H_27_N_3_NaO_2_S, 408.1772).

*Methyl 3-Thiosemicarbazonyl-7-oxochenodeoxycholicate (* ***8*** *, L* ^*8*^ *).* Light yellow solid, Yield: 53.0%,* m.p.* 285-286°C; IR (KBr) *ν*/cm^−1^: 2947, 2868, 1706, 1624, 1434, 1329, 1170, 1023; ^1^H NMR (CDCl_3_, 600 MHz): 0.65 (3H, s, 18-CH_3_), 0.89 (1.2H, d, *J* = 6.6, 21-CH_3_, 3*Z*), 0.90 (1.8H, d, *J* = 6.6, 21-CH_3_, 3*E*), 1.22 (1.8H, s, 19-CH_3_, 3*E*), 1.23 (1.2H, s, 19-CH_3_, 3*Z*), 2.31 (0.6H, dd, *J* = 10.2, 5.4, C4-*β*H, 3*E*), 2.34 (0.4H, dd, *J* = 10.2, 5.4, C4-*β*H, 3*Z*), 2.84 (0.6H, dd, *J* = 13.2, 6.0, C6-*β*H, 3*E*), 2.89 (0.4H, dd, *J* = 13.2, 6.0, C6-*β*H, 3*Z*), 3.63 (3H, s, OCH_3_), 6.52 (1H, br s, -NH_2_), 7.19 (0.4H, d, *J* = 4.2, -NH_2_, 3*Z*), 7.19 (0.6H, d, *J* = 4.2, -NH_2_, 3*E*), 8.93 (0.6H, s, -NH-, 3*E*), 8.97 (0.4H, s, -NH-, 3*Z*); ^13^C NMR (CDCl_3_, 150 MHz): 211.7 (7-C, 3*E*), 211.5 (7-C, 3*Z*), 178.9 (C=S), 174.8 (24-C), 155.4 (3-C, 3*E*), 155.3 (3-C, 3*Z*), 54.9 (17-C), 51.7 (O-CH_3_), 49.6 (9-C), 49.0 (8-C, 3*E*), 48.9 (8-C, 3*Z*), 47.7 (14-C), 46.8 (5-C, 3*Z*), 45.0 (5-C, 3*E*), 43.1 (13-C), 42.8 (10-C), 42.7 (6-C), 38.9 (12-C), 36.5 (20-C), 35.9 (4-C, 3-*E*), 35.3 (4-C, 3-*Z*), 31.2 (22-C), 31.1 (23-C), 29.8 (2-C, 3*E*), 28.7 (2-C, 3*Z*), 28.4 (16-C), 24.9 (1-C, 3*E*), 24.8 (1-C, 3*Z*), 22.9 (15-C), 22.1 (11-C), 18.5 (21-C), 14.3 (19-C), 12.2 (18-C); HREIMS: *m*/*z* 476.2941 [M+H]^+^ (calcd for C_26_H_42_N_2_O_3_S, 476.2947).

*Methyl 3-Thiosemicarbazonyl-12-oxo-7-deoxycholicate (* ***13*** *, L* ^*13*^ *).* Light yellow solid, Yield: 52.5%,* m.p.* 125–127°C; IR (KBr) *ν*/cm^−1^: 3431, 1736, 1706, 1591, 1494; ^1^H NMR (CDCl_3_, 300 MHz): 0.841 (3H, d, *J* = 6.3, 21-CH_3_), 1.034 (3H, s, 19-CH_3_), 1.062 (3H, s, 18-CH_3_), 2.60–2.49 (2H, m, C11-H), 3.657 (3H, s, OCH_3_), 6.496 (1H, br s, -NH_2_), 7.214 (1H, br s, -NH_2_), 8.897 (1H, s, -NH-); ^13^C NMR (CDCl_3_, 150 MHz): 11.7 (18-C), 18.6 (19-C), 22.3 (21-C), 24.3 (15-C), 25.6 (16-C), 27.5 (1-C), 29.8 (2-C), 30.5 (6-C), 31.3 (7-C), 35.4 (22-C), 35.6 (23-C), 36.0 (4-C), 37.0 (20-C), 38.3 (11-C), 42.3 (8-C), 43.7 (10-C), 44.4 (13-C), 46.5 (5-C), 51.5 (OCH_3_), 57.5 (9-C), 58.4 (17-C), 58.5 (14-C), 156.9 (3-C), 174.6 (24-C), 178.7 (C=S), 214.3 (12-C); HREIMS: *m*/*z* 476.2942 [M+H]^+^ (calcd for C_26_H_42_N_3_O_3_S, 476.2947).

#### 2.3.2. Methyl 3-Thiosemicarbazonyl-7-hydroxychenodeoxycholicate (**10**, L^10^)

To the stirred solution of** 8** (674 mg, 1.42 mmol) in CH_3_OH (30 mL) was added NaBH_4_ (96 mg, 2.52 mmol) in 10 min at room temperature. After no starting material was observed (the progress of the reaction was monitored by TLC, petroleum ether/ethyl acetate = 1 : 1), the reaction was stopped. The solution was neutralized with 1 M HCl. After evaporation of the majority of MeOH under reduced pressure, proper water was added. The residue was extracted with ethyl acetate. The organic layer was washed with cold water, saturated NaHCO_3_ solution, and saturated brines. After drying over anhydrous sodium sulfate, the solvent was removed under reduced pressure. A crude product was chromatographed on silica gel (elution: *V*_petroleum  ether_ : *V*_ethyl  acetate_ = 2 : 1) to give 285 mg of compound** 10** (42.3%) as a white solid. IR (KBr) *ν*/cm^−1^: 1165, 1434, 1501, 1589, 1733, 2930, 3429; ^1^H NMR (CDCl_3_, 600 MHz): 0.655 (1H, s, 18-CH_3_), 0.896 (3H, d, *J* = 6.6, 21-CH_3_), 0.946 (3H, s, 19-CH_3_), 2.36–2.31 (1H, m, C23-H), 2.55–2.52 (1H, m, C23-H), 2.894 (1H, br s, OH), 3.060 (1H, dd, *J* = 15.6, 13.2, C2-*β*H), 3.636 (3H, s, OCH_3_), 3.932 (1H, br s, C7-H), 6.309 (1H, d, *J* = 4.2, -NH_2_), 7.281 (1H, d, *J* = 4.2, -NH_2_), 9.135 (1H, s, -NH-); ^13^C NMR (CDCl_3_, 150 MHz): 11.9 (18-C), 18.4 (19-C), 21.2 (21-C), 22.4 (11-C), 23.8 (15-C), 28.2 (1-C), 30.2 (2-C), 31.0 (16-C), 31.1 (23-C), 31.2 (22-C), 33.2 (4-C), 33.7 (20-C), 35.5 (6-C), 35.9 (12-C), 37.4 (10-C), 39.6 (8-C), 39.7 (9-C), 42.3 (13-C), 42.8 (5-C), 50.3 (14-C), 51.6 (-OCH_3_), 55.9 (17-C), 68.7 (7-C), 158.7 (3-C), 174.9 (24-C), 178.4 (C=S); HREIMS: *m*/*z* 478.3108 [M+H]^+^ (calcd for C_26_H_44_N_3_O_3_S, 478.3103).

#### 2.3.3. Preparation of Platinum(II) Complexes

Solution of steroidal thiosemicarbazone (0.1 mmol) in methanol (8 mL) and 0.1 mmol K_2_PtCl_4_ was added in the round bottom flask. The mixture was stirred for 5 hours at 70°C. The reaction was terminated when large precipitant was emerged. The resulting suspension was filtered, washed with ethyl acetate and water, and dried in a desiccator over phosphorus pentoxide to give target products.

*[PtL* ^*2*^ *Cl*_*2*_ *] (Compound* ***3*** *).* Light yellow solid, Yield: 48.5%;* m.p.* 297–299°C; IR (KBr) *ν*/cm^−1^: 3416, 2927, 1609, 1584, 1499, 1409, 1160, 878; ^1^H NMR (300 MHz, DMSO) *δ*: 0.90 (3H, s, 18-CH_3_), 0.94 (0.9H, s, 18-CH_3_), 2.77–2.57 (2H, m, C16-H), 6.46 (1H, d, *J* = 2.4, C4-H), 6.52 (1H, dd, *J* = 8.4, 2.4, C2-H), 7.03 (0.31H, d, *J* = 9.0, C1-H), 7.07 (0.69H, d, *J* = 8.4, C1-H), 8.38 (1H, s, -NH_2_), 8.99 (1H, s, -NH_2_), 10.77 (1H, s, -NH-); ^13^C NMR (75 MHz, DMSO) *δ*: 12.5 (18-C), 14.2 (18-C), 20.9 (11-C), 23.6 (15-C), 26.3 (16-C), 26.8 (7-C), 29.1 (6-C), 31.8 (16-C), 35.2 (12-C), 35.4 (12-C), 37.5 (9-C), 40.1 (8-C), 43.1 (13-C), 49.4 (13-C), 53.6 (14-C), 59.9 (14-C), 112.9 (2-C), 115.0 (4-C), 126.0 (1-C), 130.1 (10-C), 137.1 (5-C), 155.1 (3-C), 170.5 (17-C), 185.9 (C=S).; HREIMS: *m*/*z* 607.0728 [M+H]^+^ (calcd for C_19_H_26_Cl_2_N_3_OPtS, 607.0665).

*[PtL* ^*5*^ *Cl*_*2*_ *] (Compound* ***6*** *).* Yellow solid, Yield: 51.2%;* m.p.* 287-288°C; IR (KBr) *ν*/cm^−1^: 3329, 3129, 1763, 1626, 1556, 1489, 1202, 941, 811; ^1^H NMR (600 MHz, DMSO) *δ*: 0.91 (3H, s, 18-CH_3_), 2.23 (3H, s, COCH_3_), 2.63 (1H, dd, *J* = 19.2, 9.0, C6-H), 2.84-2.81 (2H, m, C16-H), 6.80 (1H, d, *J* = 2.4, C4-H), 6.85 (1H, dd, *J* = 8.4, 2.4, C2-H), 7.32 (1H, d, *J* = 8.4, C1-H), 8.40 (1H, s, -NH_2_), 8.99 (1H, s, -NH_2_), 10.78 (1H, s, -NH-); HREIMS: [M+H]^+^ 619.1162 (calcd for C_21_H_27_KN_3_O_2_PtS, 619.1109).

*[PtL* ^*8*^ *Cl*_*2*_ *] (Compound* ***9*** *).* Light yellow solid, Yield: 46.7%,* m.p.* 270–272°C; IR (KBr) *ν*/cm^−1^: 1706, 1624, 1437, 1329; ^1^H NMR (CDCl_3_, 600 MHz): 0.62 (3H, s, 18-CH_3_), 0.87 (3H, d, *J* = 6.6, 21-CH_3_), 1.17 (1.8H, s, 19-CH_3_, R-), 1.20 (1.2H, s, 19-CH_3_, S-), 2.91 (0.4H, dd, *J* = 12.6, 4.8, C6-*β*H, S-), 2.96 (0.6H, dd, *J* = 12.6, 6.0, C6-*β*H, R-), 3.57 (3H, s, OCH_3_), 6.79 (1H, s, -NH_2_), 6.84 (1H, s, -NH_2_); ^13^C NMR (CDCl_3_, 150 MHz): 11.9 (18-C), 18.3 (19-C), 21.6 (21), 21.9 (11-C), 22.0 (1-C), 24.4 (15-C), 27.9 (16-C), 30.4 (2-C), 30.7 (23-C), 34.8 (22-C), 35.2 (4-C, S-), 35.5 (4-C, R-), 36.3 (20-C), 38.3 (12-C), 42.1 (10-C), 42.23 (6-C, S-), 42.29 (6-C, R-), 42.7 (13-C), 44.0 (5-C, S-), 44.6 (5-C, R-), 46.3 (14-C), 48.4 (8-C,* S-*), 48.6 (8-C,* R-*), 49.1 (9-C), 51.3 (OCH_3_), 54.3 (17-C), 168.0 (3-C,* R-*), 168.1 (3-C,* S-*), 173.8 (24-C), 175.4 (C=S,* S-*), 176.0 (C=S,* R-*), 211.3 (7-C); HREIMS: *m*/*z* 669.2430 [M]^+^ (calcd for C_26_H_40_N_3_O_3_PtS, 669.2438).

*[PtL* ^*10*^ *Cl*_*2*_ *] (Compound* ***11*** *).* Light yellow solid, Yield: 51.1%,* m.p.* 272–274°C; IR (KBr) *ν*/cm^−1^: 3434, 2932, 1733, 1629, 1437, 1329; ^1^H NMR (DMSO, 600 MHz): 0.62 (2.1H, s, 18-CH_3_,* R-*), 0.63 (0.9H, s, 18-CH_3_,* S-*), 0.88 (2.1H, s, 19-CH_3_,* R-*), 0.89 (3H, d, *J* = 6.6, 21-CH_3_), 0.93 (0.9H, s, 19-CH_3_,* S-*), 2.23–2.16 (1H, m, C23-H), 2.35–2.30 (1H, m, C23-H), 3.57 (3H, s, OCH_3_), 3.69–3.61 (1H, m, C7-H), 6.62 (0.3H, s, -NH_2_,* S-*), 6.50 (0.3H, s, -NH_2_,* S-*), 6.70 (0.7H, s, -NH_2_,* R-*), 6.76 (0.7H, s, -NH_2_,* R-*); ^13^C NMR (DMSO, 150 MHz): 11.7 (18-C), 18.2 (19-C), 20.6 (21-C), 21.9 (11-C), 23.2 (15-C), 27.8 (1-C), 30.4 (2-C), 30.7 (16-C), 32.1 (23-C), 32.9 (22-C), 33.7 (4-C,* S-*), 33.9 (4-C,* R-*), 34.4 (20-C 5-C), 34.9 (6-C), 35.4 (12-C), 37.8 (10-C), 40.5 (8-C), 42.0 (9-C), 42.1 (13-C), 45.2 (5-C), 49.9 (14-C), 51.3 (OCH_3_), 55.5 (17-C), 66.2 (7-C), 167.4 (3-C,* S-*), 167.8 (3-C,* R-*), 173.9 (24-C), 177.8 (C=S,* R-*), 178.4 (C=S,* S-*); HREIMS: *m*/*z* 695.2554 [M+Na]^+^ (calcd for C_26_H_43_N_3_NaO_3_PtS, 695.2570).

*[PtL* ^*13*^ *Cl*_*2*_ *] (Compound* ***14*** *).* Light yellow solid, Yield: 41.0%,* m.p.* 270–272°C; IR (KBr) *ν*/cm^−1^: 3426, 1736, 1706, 1616, 1447; ^1^H NMR (DMSO, 600 MHz): 0.75 (1.4H, s, 18-CH_3_,* S-*), 0.76 (1.6H, s, 18-CH_3_,* R-*), 0.97 (3H, s, 19-CH_3_), 1.00 (3H, d, *J* = 7.2, 21-CH_3_), 2.78–2.68 (1H, m, C20-H), 3.19 (0.46H, d, *J* = 13.2, C2-H, S-), 3.46 (0.54H, d, *J* = 15.0, C4-H, R-), 3.57 (3H, s, OCH_3_), 6.75 (1H, br s, -NH_2_), 6.79 (1H, br s, -NH_2_); ^13^C NMR (DMSO, 150 MHz): 11.3 (18-C), 18.6 (19-C), 23.9 (21-C), 25.0 (15-C), 26.3 (16-C), 27.1 (1-C), 30.3 (2-C), 30.7 (6-C), 35.1 (7-C), 35.4 (22-C), 35.7 (23-C), 36.5 (4-C,* S-*), 36.7 (4-C,* R-*), 37.3 (20-C), 38.0 (11-C), 42.8 (8-C), 43.5 (10-C), 45.4 (13-C), 46.3 (5-C), 51.3 (OCH_3_), 56.8 (9-C), 57.3 (17-C), 57.4 (14-C), 167.7 (3-C,* S-*), 168.1 (3-C,* R-*), 173.7 (24-C), 177.0 (C=S,* S-*), 177.6 (C=S,* R-*), 211.5 (12-C,* S-*), 213.4 (12-C,* R-*); HREIMS: *m*/*z* 671.2555 [M+H]^+^ (calcd for C_26_H_42_N_3_O_3_PtS, 671.2595).

### 2.4. Cytotoxicity Assay In Vitro

The antiproliferative activity of all steroidal thiosemicarbazones and their Pt(II) metal complexes and cisplatin on Bel 7404 (human liver carcinoma), HeLa (human cervical carcinoma), and HEK293T (normal kidney epithelial cells) cell lines was determined by using the MTT method. The detailed procedure has been reported in our previous work [24].

## 3. Results and Discussion

### 3.1. Synthesis and Characterization

**Scheme 1:**
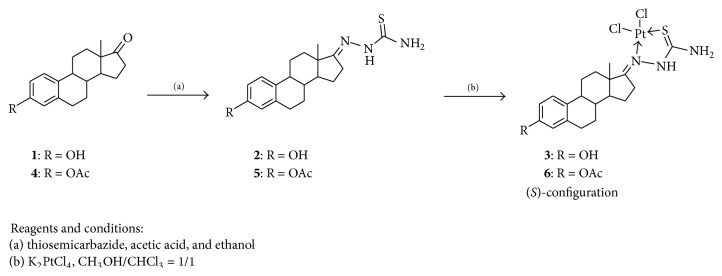
Synthesis of estrone-17-thiosemicarbazone platinum(II).

The synthetic route and the structures of complexes** 3** and** 6** were outlined in Scheme 1. Steroidal thiosemicarbazones** 2** and** 5** were obtained as a (*E*)-configuration by reacting estrone with thiosemicarbazide, and the reaction of compounds** 2** and** 5** with K_2_PtCl_4_ gave steroidal platinum (Pt(II)) complexes** 3** and** 6** as (*S*)-configuration, respectively. The structures of** 3** and** 6** were confirmed by analysis of UV, IR, NMR, and HRMS.

**Scheme 2:**
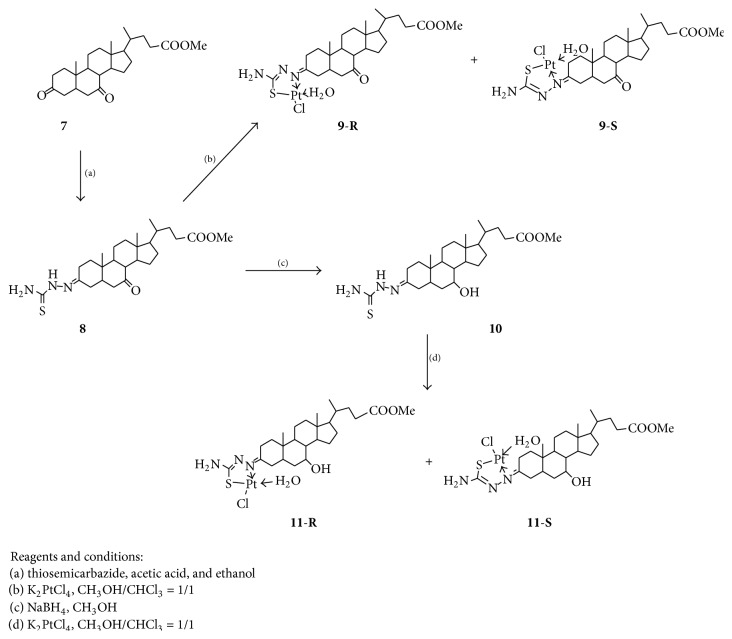
Synthesis of methyl chenodeoxycholate complexes** 9** and** 10**.

In order to investigate the effect of position of pharmacophore on the antiproliferative activity of complexes, we prepared complexes** 9** and** 11** using chenodeoxycholic acid as a starting material (Scheme 2). Starting from compound** 7**, compound** 8** with 3-thiosemicarbazone group was yielded as a mixture of (3*E*)- and (3*Z*)-isomer (ratio: 0.6 : 0.4, ^1^H NMR data) by controlling an appropriate molar ratio of** 7** and thiosemicarbazide because 3-carbonyl group was more active than 7-carbonyl, and thiosemicarbazide was selectively reacted with 3-carbonyl. The reaction of compound** 8** with K_2_PtCl_4_ afforded further complex** 9** as a mixture of (*R*)- and (*S*)-configuration isomer (**9**-**R** :** 9**-**S** = 3 : 2, ^1^H NMR data).

Next, the 7-carbonyl of** 8** was converted to 7-hydroxyl of compound** 10** by the reduction of NaBH_4_, but the 3-thiosemicarbazone group was still kept in compound** 10**. The reaction of** 10** with K_2_PtCl_4_ gave complex** 11**. Complex** 11** was a mixture of (*R*)- and (*S*)-configuration isomer also (**9**-**R** :** 9**-**S** = 7 : 3, ^1^H NMR data).

The structures of complexes** 9** and** 11** had been determined by analysis of IR, UV, NMR, and HRMS.

**Scheme 3:**
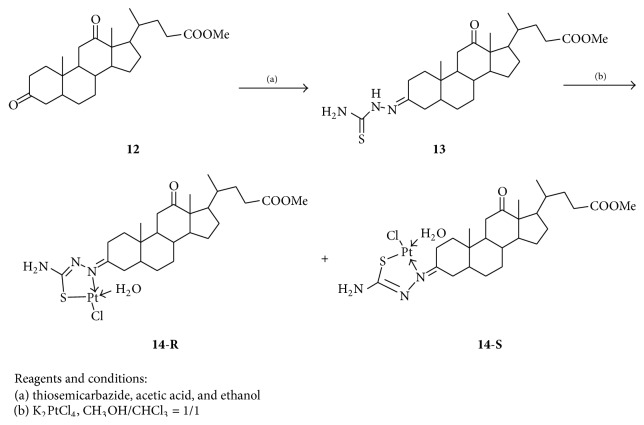
Synthesis of methyl 7-deoxycholate complex** 14**.

Using 7-deoxycholic acid as a starting material, another steroidal thiosemicarbazone platinum (Pt(II)) complex** 14** was synthesized (Scheme 3). Similarly, complex** 14** was a mixture of (*R*)- and (*S*)-configuration isomer (**14**-**R** :** 14**-**S** = 0.54 : 0.46, ^1^H NMR data) and their structures had been confirmed by analysis of IR, UV, NMR, and HRMS.

### 3.2. Cytotoxic Activity In Vitro

The antiproliferative activities of all steroidal thiosemicarbazones and their Pt(II) metal complexes were determined in vitro on Bel 7404, HeLa, and HEK293T. The MTT method was used to assay the antiproliferative activity and cisplatin was used as a positive control. The results are summarized as IC_50_ values in *μ*M in Table 1.

As shown in Table 1, steroidal platinum (Pt(II)) complexes** 9**,** 11**, and** 14** are almost inactive against Bel 7404 and HeLa cells. However, complexes** 3** and** 6** exhibited an obvious cytotoxicity to HeLa and Bel 7404 cells. In particular, complex** 6** showed an excellent antiproliferative activity against HeLa cells with the IC_50_ values of 9.2 *μ*M and had a better cytotoxicity compared with its precursor.

Comparing the antiproliferative activity of estrone-17-thiosemicarbazone platinum(II) with that of methyl chenodeoxycholate-thiosemicarbazone platinum(II) and methyl 7-deoxycholate-thiosemicarbazone platinum(II), we can see that estrone-17-thiosemicarbazone platinum(II) shows a better inhibiting activity than methyl chenodeoxycholate-thiosemicarbazone platinum(II) and methyl 7-deoxycholate-thiosemicarbazone platinum(II). A reason is that estrone-17-thiosemicarbazone platinum(II) with the structure of steroidal nucleus of estrone may be connected with the metabolism of estrogen.

Here, the positive control cisplatin and complex** 6** displayed similar cytotoxicity against HeLa cells but cisplatin had obvious cytotoxicity to normal kidney epithelial cells HEK293T and complex** 6** was almost inactive.

The Selectivity Index (SI) was defined as the ratio of the cytotoxicity of a compound with respect to normal cells (IC_50_ HEK293T) versus cancer cells and used to determine the criterion of effectiveness of the compounds. The SI values of the complexes are listed in Table 2.

One important criterion for a therapeutic drug for cancer is to have minimal or no side effects to normal body cells of patients undergoing chemotherapy. Considering that a higher SI corresponds to greater overall anticancer activity, we can affirm that complex** 6** is an excellent selective inhibitor against HeLa cells, which deserve further study (SI value: complex** 6** 21.7, cisplatin 1.0).

## 4. Conclusion

In conclusion, we had prepared some steroidal thiosemicarbazone platinum(II) complexes and assayed their antiproliferative activities. The results showed that estrone-17-thiosemicarbazone platinum(II) displayed a better inhibiting activity than methyl chenodeoxycholate-thiosemicarbazone platinum(II) and methyl 7-deoxycholate- thiosemicarbazone platinum(II). Among them, complex** 6** based on the structure of estrone was found to be a valuable selective inhibitor against HeLa cells possessing the IC_50_ values of 9.2 *μ*M and SI value of 21.7. The result may be useful for the design of novel chemotherapeutic drugs.

## Figures and Tables

**Table 1:** Cytotoxicity^a^ of steroidal thiosemicarbazones and their Pt-complexes in vitro (IC_50_: *µ*M)^b^.

Compounds	Bel-7404	HeLa	HEK293T
**2**	19	34	ND
**3**	39	23	>200
**5**	>200	42	>200
**6**	86	9.2	>200
**8**	>200	96	>200
**9**	>200	156	>200
**10**	>200	142	>200
**11**	>200	>200	>200
**13**	>200	>200	>200
**14**	103	102	>200
Cisplatin	23.2	10.1	10.3

^a^Cytotoxicity as IC_50_ for each cell line is the concentration of compound which reduced by 50% the optical density of treated cells with respect to untreated cells using the MTT assay.

^b^Data represent the mean values of three independent determinations.

**Table 2:** SI values of steroidal thiosemicarbazone Pt-complexes.

Compounds	**3**	**6**	**9**	**11**	**14**	Cisplatin
SI_Bel 7404_	5.1	2.3	—	—	1.9	—
SI_HeLa_	8.7	21.7	1.3	—	2.0	1.0
